# Cryobiopsies are diagnostic in Pleuroparenchymal and Airway-centered Fibroelastosis

**DOI:** 10.1186/s12931-018-0839-3

**Published:** 2018-07-13

**Authors:** Sissel Kronborg-White, Claudia Ravaglia, Alessandra Dubini, Sara Piciucchi, Sara Tomassetti, Elisabeth Bendstrup, Venerino Poletti

**Affiliations:** 1 Department of Respiratory Diseases and Allergy, Aarhus University Hospital, Aarhus, Denmark; 2 Department of the Diseases of the Thorax, Ospedale Morgagni, Forli, Italy; 3 Department of Pathology, Ospedale Morgagni, Forli, Italy; 4 Department of Radiology, Ospedale Morgagni, Forli, Italy

**Keywords:** Cryobiopsy, Pleuroparenchymal fibroelastosis, Airway-centered fibroelastosis

## Abstract

**Background:**

Idiopathic pulmonary fibroelastosis (iPPFE) is a rare lung lesion characterized by pleural and subpleural parenchymal thickening due to accumulation of fibroelastotic tissue. Only recently, a few cases with a peribronchiolar distribution of fibroelastotic tissue have been reported. These lesions are more prominent in the upper lobes. Even though high resolution computed tomography (HRCT) scan features are considered characteristic, a histological confirmation is suggested, mainly when the clinical setting is not clearly defined. However, due to non-negligible complications, a surgical lung biopsy is not often recommended. The prognosis is usually poor and currently, the only effective treatment is lung transplantation.

**Method:**

Patients with a multidisciplinary diagnosis of iPPFE or airway-centered fibroelastosis (airway-centered FE), with histological confirmation by transbronchial cryobiopsy, were identified from an ongoing interstitial lung disease registry. Data on patient demographics, HRCT patterns, size and number of biopsies, histology patterns and complications were registered.

**Results:**

Seven patients were diagnosed with iPPFE and one patient was diagnosed with airway-centered FE. Pneumothorax was documented in three cases, but none of them required a chest tube. No other complications during or after the procedure were observed.

**Conclusion:**

This study suggests that using cryobiopsies in the diagnostics of PPFE and airway-centered FE is safe and effective.

## Background

Transbronchial cryobiopsies (cTBB) has emerged as a new method for obtaining lung tissue samples in the diagnostics of fibrosing interstitial lung diseases (ILD) [1–4]. Surgical lung biopsies (SLB) has been used for a long time in the diagnosis of this heterogeneous group of diseases due to larger samples and less artefacts compared to forceps transbronchial biopsy [5]. The procedure is associated with a high morbidity and a non-negligible mortality rate [1]. cTBB has been shown to be a safer procedure a with lower mortality and complication rate. The diagnostic yield is about 80% [6–8].

Idiopathic pleuroparenchymal fibroelastosis (iPPFE) is a rare form of idiopathic interstitial pneumonia, recently recognized as a new entity [5, 9, 10]. Until now, a little more than 100 cases have been reported [11]. iPPFE is characterized by fibrosis involving the visceral pleura and the subpleural lung parenchyma with an upper lobe predominance [9]. The fibrosing process is elastotic and associated with intra-alveolar fibrosis and scattered fibroblastic foci [12]. A significant increase of lymphatic vessels and podoplanin-positive myofibroblasts seem to be specific histological and immunohistochemical markers of this pathologic entity [13, 14].

This histological pattern has been reported in association with drugs, infections, hypersensitivity pneumonitis, lung and bone marrow transplantation and autoimmunity. When no inciting cause or specific clinical setting is identifiable, the disorder is labeled as idiopathic [15–17]. Patients with iPPFE have a median age of approximately 53 years (range 13–87) with no gender predilection. The symptoms are shortness of breath, cough, weight loss, relapsing low grade fever and chest pain [18]. Pulmonary function tests (PFT) are impaired with a restrictive pattern although a few patients have presented with obstruction [19, 20]. High-resolution computed tomography (HRCT) shows pleuroparenchymal thickening, traction bronchiectasis in the apical region and often upper lobe volume loss [16, 17]. The differential diagnosis includes amongst others advanced hypersensitivity pneumonia, advanced sarcoidosis, advanced smoking-related ILD, asbestosis, and mesothelioma. The prognosis varies with some patients relentlessly progressing while others have a more prolonged and stable course of disease [11].

A new entity of airway-centered fibroelastosis (airway-centered FE) has recently been described in five non-smoking middle-aged women; previous history of chronic asthma was confirmed in four of the women. Airway-centered FE is characterized by fibroelastotic lesions very similar to that observed in PPFE but centered mainly around the airways. [21]. Unlike patients with PPFE, patients with airway-centered FE experienced acute exacerbations with dyspnea and wheezing. PFTs show both obstructive and restrictive patterns [21]. It is difficult to define the relationship between the two entities mainly because of the rarity of airways-centered FE. However, due to the similar histopathologic changes (accumulation of characteristic fibroelastotic tissue) we include both disorders in this case series.

As patients with iPPFE and airway-centered FE have stiffened lung parenchyma predominantly in the upper lobes, SLB is often considered too risky. In this context, cTBB could represent a valid alternative to confirm the histological data with the clinical-radiological hypothesis [19]. In this study, we present a cohort of patients identified at one ILD referral center where the morphological diagnosis of fibroelastotic lung disease was obtained by cTBB.

## Methods

### Patients

Patients were identified from an ongoing ILD registry at the Department of Diseases of the Thorax at Forlì from January 2010 to March 2017. Only patients diagnosed with iPPFE or airway-centered FE after a multidisciplinary team conference were enrolled in the study. We excluded from the study: 1) patients with a multidisciplinary diagnosis of PPFE, but without available histology, 2) patients with histological features confirmed by cTBB of PPFE in the upper lobes + UIP/NSIP/DIP in the lower lobes obtaining a final multidisciplinary diagnosis of IPF or unclassifiable ILD according to recent papers [22] and 3) patients with a diagnosis of secondary fibroelastotic lung disease (related to drugs, collagen vascular disorders, transplantation or bronchiolar infection). Data on gender, age, PFT (forced vital capacity (FVC) and diffusing capacity of the lung for carbon monoxide (DLCO)), exposure, comorbidities, radiological and pathological findings were retrieved from the registries. The indication for cTBB was a formal multidisciplinary decision made by the physicians evaluating the patients. Each case was discussed during the weekly meeting in a multidisciplinary setting with the participation of pulmonologists, radiologists, and pathologists. After discussion, a definite diagnosis was suggested. All cases were investigated for features of infection (in particular cultures for *mycobacteria* and *aspergillus*), autoimmunity, as well as family history and all patients received bronchial/bronchoalveolar lavage (BAL).

### Bronchoscopic cryobiopsies

The bronchoscopic cryobiopsy procedure has previously been described in details [1]: patients were intubated with a rigid tracheoscope under deep sedation and cTBB was performed using a flexible cryoprobe (2.4 mm or 1.9 mm, ERBE, Tubingen, Germany) under fluoroscopic guidance. The biopsy site was decided after considering the HRCT scan features.

### High-resolution computed tomography (HRCT)

HRCT images performed within 1 month prior to the procedure were reviewed by an expert radiologist (S.P). One or 1.5 mm collimation sections were obtained at 10 mm intervals, or volumetrically on multidetector CT scanners with 0.6 or 1 mm collimation and 1 mm reconstruction; all images were reviewed using window settings optimized for lung parenchyma (width: 1.500–1.600 HU; level: 2.500–2.600 HU).

### Pathologic interpretations

Before the multidisciplinary meeting, two pathologists (AD and VP) independently examined the pathological specimens. Consensus was reached concerning the final common pathologic interpretation for each case. Elastic fibers within fibrosis were confirmed by using specific stains (Verhoeff-van Gieson procedure) [18]. The pathologic diagnosis of fibroelastotic disease was made according to the presence of fibrous thickening of the visceral pleura, homogeneous, dense, intra-alveolar fibrosis with subpleural/septal elastosis sharply separated from the adjacent “spared” lung parenchyma (PPFE) or according to the presence of peribronchiolar/centroacinar dense fibrotic areas containing mainly elastic fibers (airway-centered FE).

## Results

**Fig. 1:**
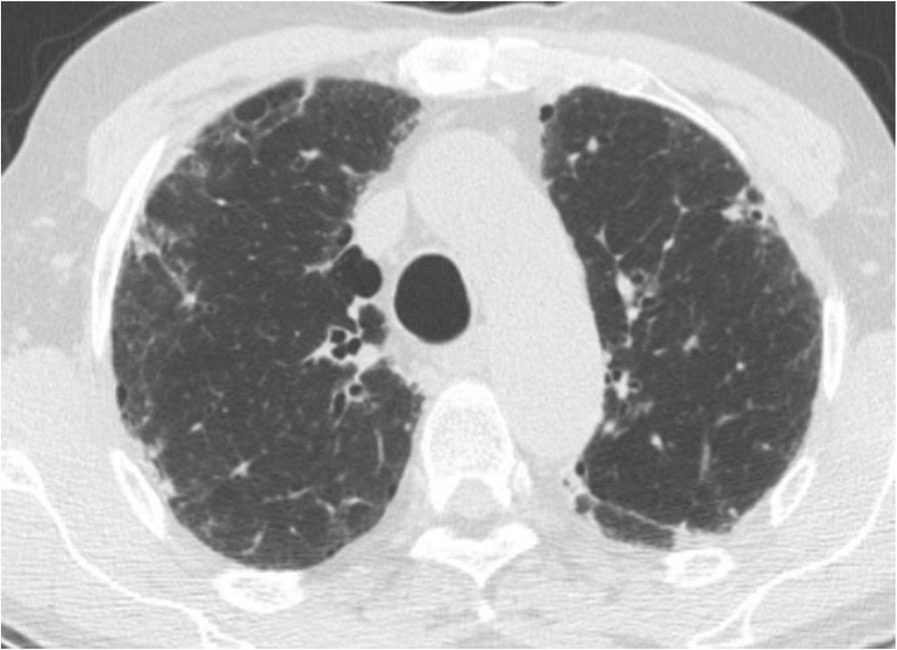
High resolution CT scan of one patient affected by PPFE, showing severe pleural and sub-pleural thickening with moderate fibrotic changes in the marginal parenchyma

**Fig. 2:**
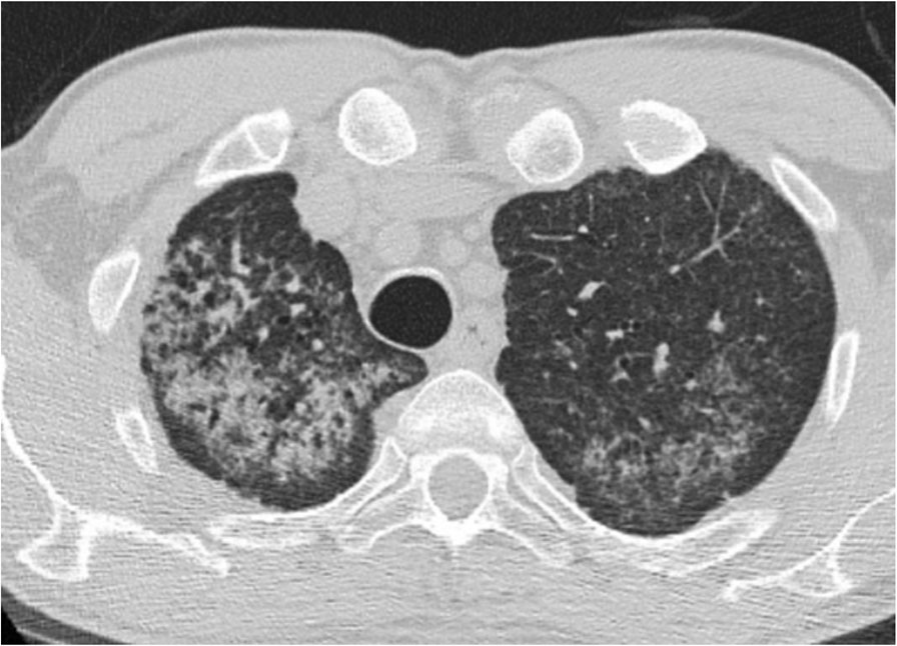
High resolution CT scan showing extensive peribronchiolar consolidations, almost exclusively involving the upper zones of the lungs (mainly the right upper lobe). The pleura and subpleural parenchyma is almost always spared

Seven patients (6 females/1 male) were diagnosed with iPPFE and 1 male was diagnosed with airway-centered FE between 2010 and 2017 (Table 1). Age at diagnosis ranged from 40 to 65 years. Mean PFT showed a FVC of 71% and a DLCO of 65%. All iPPFE patients were never-smokers while the patient with airway-centered FE was a former smoker (20 pack years). Cough or exertional dyspnea was a significant onset symptom in all patients; three patients also complained of chest pain and fever was present in three patients with iPPFE. HRCT in the patients with iPPFE was characterized by pleural thickening with associated sub-pleural fibrosis, lung volume loss, distortion of the lung architecture with multiple traction bronchiectasis and/or honeycombing and some ground glass opacities with upper lobe predominance; signs of parenchymal lung injury in the lower lobes were absent (Fig. 1). The HRCT scan of the patient with airway-centered FE was characterized by alveolar consolidation centered around bronchi/bronchioles with sparing of the subpleural regions with upper lobe predominance (Fig. 2).

**Table 1:** Clinical data

	Patient 1	Patient 2	Patient 3	Patient 4	Patient 5	Patient 6	Patient 7	Patient 8
Age (y)	65	57	62	55	65	43	40	55
Final diagnosis	iPPFE	iPPFE	iPPFE	iPPFE	iPPFE	iPPFE	iPPFE	ACFE
Smoke (Y/N/ex)	N	N	N	N	N	N	N	Ex
Sex (M/F)	F	F	F	F	F	M	F	M
Symptoms	chest pain, cough	fever, cough	fever, cough	weight loss, dyspnea	fever, chest pain and dyspnea	weight loss, dyspnea	dyspnea	Chest pain, dyspnea
FVC %	80	75	61	62	79	58	94	62
DLCO %	74	64	55	51	72	69	74	64

Abbreviations: *iPPFE* = idiopathic pleuro-parenchymal fibroelastosis; *ACFE* = airway centered fibroelastosis

Differential cell counts from BAL were as follows: mean neutrophils 31%, lymphocytes 19%, eosinophils 0.3% and macrophages 50%. BAL cultures were negative for bacteria, fungi and mycobacteria in all cases.

In seven patients, the 2.4 probe was used. In one patient, a 1.9 cryoprobe was used because of excessive resistance during retrieval of the 2.4 probe, possibly due to bronchomalacia. The freezing time was six seconds for the 2.4 probe and eight seconds for the 1.9 probe. Regarding safety, no bleeding (mild, moderate or severe) was observed. Pneumothorax was documented in three cases, but none of them required insertion of a chest tube. No death, acute lung injury, persistent fever, prolonged air leak, pneumonia/empyema, or other adverse events were observed after cTBB in any of the patients (Table 2).

**Table 2:** Cryobiopsy data

	Patient 1	Patient 2	Patient 3	Patient 4	Patient 5	Patient 6	Patient 7	Patient 8
Site 1	RUL (post)	RUL (post)	Lingula	RUL	RUL (ant)	RUL	LUL	RUL
Histology site 1	PPFE with nodular lymphoid aggregates	PPFE features	PPFE features	PPFE features	PPFE features	PPFE features with poorly formed granulomas and ossification	PPFE features	Peribronchiolar fibroelastosis with mild pleuro-parenchymal elastosis
Site 2	RUL (ant)	RUL (ant)	LUL		RUL (post)			
Histology site 2	Normal pleura and parenchyma	PPFE features	PPFE features		PPFE features			
Pnx	Y	N	Y	N	Y	N	N	N
Drainage	N		N		N			
Other complications	N	N	N	N	N	N	N	N
BAL (%)	Y	Y	Y	Y	Y	Y	Y	Y
-macrophages	57	6	49	53	78	NA	56	NA
-neutrophils	35	92	40	7	10		2	
-lymphocytes	8	2	10	40	11		42	
-eosinophils	0	0	1	0	1		0	

Abbreviations: *RUL* = right upper lobe, *LUL* = left upper lobe, *pnx* = pneumothorax, *BAL* = bronchoalveolar lavage

In four patients, biopsies were carried out in the same segment and in four patients, different segments in the upper lobe were biopsIed. Visceral pleura was detected in five out of eight cases.

**Fig. 3:**
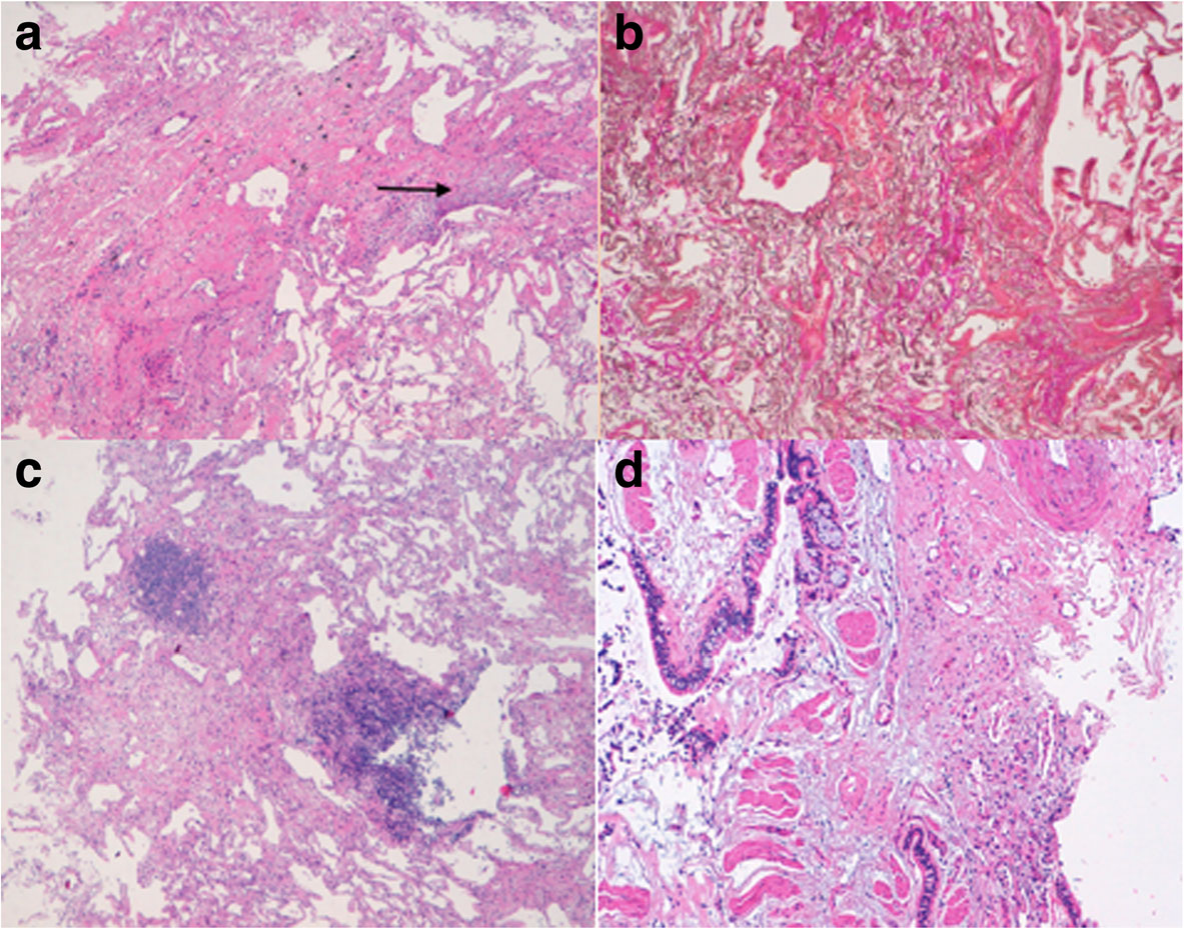
(Top: PPFE left: **a** right: **b** lower: airway-centered FE left: **c** right: **d**). **a** Markedly thickened visceral pleura and prominent sub pleural fibrosis comprised of a homogenous mixture of elastic tissue and dense collagen. Some fibroblastic foci are evident at the edge between the fibrotic area and normal lung parenchyma (H&E, low power). **b** The elastic tissue is clearly marked by a specific stain (Verhoeff-van Gieson stain, mid power). **c**. Fibroelastotic tissue sited in the peribronchiolar acinar area with constructive bronchiolitis (only pulmonary artery branched are identifiable) and focal nodular lymphocytic inflammation. The rest of the lung parenchyma is spared. (H&E, low power). **d** Another sample showing larger airways with goblet cell metaplasia, smooth muscle hypertrophia surrounded by fibroelastotic tissue. (H&E, mid power)

All specimens were considered adequate, as they contained alveolar lung tissue. Histological features characteristic for PPFE were found in all seven patients with iPPFE; these included increased elastic fibers with septal elastosis in the sub-pleural parenchyma, intra-alveolar collagenosis and collagenous thickening of visceral pleura (Fig. 3a and b). Fibroblastic foci were identified in two patients. The specimens of the patient with airway-centered FE were characterized by peribronchiolar fibroelastosis (Fig. 3c and d). Due to the rarity of the entity, in the patient with airway-centered FE, the histological diagnosis was also confirmed by SLB.

## Discussion

Idiopathic fibroelastotic lung diseases (including iPPFE and airway-centered FE) are rare entities with a little more than 100 cases reported in the literature. The pathogenic events are not yet clarified [7, 16, 21–25].

In all the published literature case series of PPFE, most diagnoses were made by SLB [23–30]. However, SLB is associated with significant morbidity and mortality and also considerable costs [31–36]. Regarding PPFE in particular, in addition to the common complications of the procedure, post-operative bronchopleural fistulas, spontaneous, iatrogenic or chronic pneumothoraces have been reported [37, 38]. Watanabe et al. reported a case with prolonged air leak and prolonged pneumothorax, lasting two months after surgery [38]. Becker et al. reported one patient dying after SLB complicated by a large bronchopleural fistulae [37]. CT-guided transthoracic core lung biopsy has been proposed as a diagnostic tool [23], but there are still few reports regarding diagnostic yield and complications and it seems difficult with this procedure to collect more samples from different segments during the same procedure.

Airways-centered FE has been reported in five cases so far [21]. Because of its rarity the clear clinical-radiologic profile is not yet defined. The patient reported here did not have asthma and the radiological features were mainly characterized by alveolar consolidation around centriacinar regions mimicking sarcoidosis. In the paper by Pradere P et al., SLB confirmed the diagnosis with no complications [21]. However, cTBB can easily reach the peribronchiolar areas and appears more preferable. In fact, in the case included in this series small altered airways surrounded by fibroelastotic tissue were evident in the cryosamples.

The major risks after cryobiopsy are pneumothorax and bleeding. Pneumothorax may occur in approximately 10% of the cases [3, 4, 6, 39] and the risk of pneumothorax increases with a UIP pattern on histology, fibrotic reticulation on HRCT and biopsies taken close to the pleura [6, 40]. Due to the rarity of the fibroelastotic diseases, it is unknown if the complication rate is increased in these entities**.** Bleeding is common during cryobiopsy, but it is generally controlled endoscopically by using bronchial blockers and/or rigid bronchoscopy/tracheal tubes. Mortality due to acute exacerbations of idiopathic interstitial pneumonias after cryobiopsy has been reported in a few cases [40, 41].

In this paper, we have shown that cTBB is a valid method to obtain a histopathologic diagnosis in patients with suspected iPPFE and airway-centered FE and that it can be obtained with an acceptable complication rate. In fact, no bleeding (mild, moderate or severe) was observed and pneumothorax was documented in three out of eight cases, none of them requiring drainage or lasting more than 3 days. No death, acute lung injury, persistent fever, prolonged air leak, pneumonia/empyema or other adverse events occurred after cTBB in any of the cases.

Regarding histology, all specimens were considered adequate as they contained alveolar tissue and histological features characteristic for PPFE or airways-centered FE (Table 2). An interesting study that investigates the use of urinary desmosines as a noninvasive diagnostic biomarker in patients with PPFE has recently been performed [42]. Levels of urinary desmosines were significantly elevated in patients with biopsy-proven PPFE compared to patients with COPD, IPF and healthy controls. However, this biomarker needs to be validated as a diagnostic test in a large cohort of patients.

## Conclusion

In conclusion, PPFE and airway-centered FE are rare, though important differential diagnoses to other interstitial lung diseases with predilection for the upper lobes. In many cases, histology is not available due to the increased risk of a SLB. Our experiences in diagnosing iPPFE and airway-centered FE using cTBB are reported here. Complications are acceptable and less than what observed after SLB. Obtaining histological tissue may result in the unveiling of pathophysiologic mechanisms and hopefully tailored treatment in the future.
